# Predictors of posttraumatic stress disorder among displaced families in Lebanon: A cross-sectional study

**DOI:** 10.1371/journal.pgph.0005280

**Published:** 2025-10-30

**Authors:** Paula Hage Boutros, Samah Hachem, Bachir Atallah, Malak Awada, Rola Bou Serhal

**Affiliations:** 1 Department of Nutrition, Faculty of Health Sciences, Modern University for Business and Science, Ramiz Sarkis, Beirut, Lebanon; 2 Department of Public Health, Faculty of Health Sciences, Modern University for Business and Science, Ramiz Sarkis, Beirut, Lebanon; 3 Department of Optometry and Vision Sciences, Faculty of Health Sciences, Modern University for Business and Science, Ramiz Sarkis, Beirut, Lebanon; 4 Research Department, Modern University for Business and Science, Ramiz Sarkis, Beirut, Lebanon; 5 Department of Nursing, Faculty of Health Sciences, Modern University for Business and Science, Ramiz Sarkis, Beirut, Lebanon; University of California Irvine, UNITED STATES OF AMERICA

## Abstract

This study aimed to assess the prevalence of posttraumatic stress disorder (PTSD) among Lebanese individuals internally displaced by the recent conflict and to identify key predictors of PTSD. The findings are intended to guide targeted policy and advocacy strategies aimed at improving mental health outcomes in this vulnerable population. To assess our aim, a cross-sectional survey was conducted with 400 internally displaced individuals across Lebanon. PTSD was assessed using the PTSD Checklist for DSM-5 (PCL-5), depression using the PHQ-2, and anxiety using the GAD-2 scale. Food security status was measured using the Arab Family Food Security Scale (AFFSS). Capillary blood glucose levels were obtained via finger-prick and classified per ADA criteria. Resilience was measured using the Brief Resilience Scale (BRS). Logistic and linear regressions were used to identify predictors of PTSD status and symptom severity. **The results showed** the sample was predominantly female (74.3%), with most participants reporting low education (63.6%) and low monthly income (83.3% earning less than $700). PTSD was highly prevalent, with 64.8% screening positive (mean PCL-5 = 40.53, SD = 20.75). Depression (57.5%) and anxiety (57%) were also widespread. Strong predictors of PTSD included depression (OR = 1.303), anxiety (OR = 1.576), food insecurity (B = 0.803), and low education (OR = 2.77). Resilience (OR = 0.287; B = -8.210) and receiving psychological counseling (OR = 0.526) were protective, though only 28.3% accessed mental health services. In conclusion, the study revealed a substantial mental health burden among displaced individuals, with PTSD closely linked to food insecurity, psychological distress, and limited access to care. Urgent, tailored interventions are needed to address these vulnerabilities.

## Introduction

Forced displacement occurs when individuals are compelled to leave their homes due to natural disasters—such as floods, earthquakes, and droughts—or human-induced events, including armed conflict and political violence [1]. Whether displacement occurs internally or across borders, it is frequently associated with significant psychological distress. This is especially true when it is accompanied by violence, economic hardship, social isolation, and barriers to mental healthcare [2–7].

Lebanon has long been both a site of internal displacement and a host country for refugees, shaped by its geographic position and history of conflict [8]. The Lebanese Civil War (1975–1990), successive Israeli military operations (1978–2006), and the ongoing Syrian refugee crisis (since 2011) have left lasting effects on both local and displaced populations [9]. Exposure to pre-displacement trauma, combined with the stresses of displacement and resettlement, contributed to an elevated risk for psychiatric disorders, including posttraumatic stress disorder (PTSD), depression, and anxiety [10]. For example, following the 2006 Israeli conflict, 26% of Lebanese youth met criteria for PTSD, and 44% met criteria for at least one other psychiatric disorder [11]. A systematic review conducted in Lebanon showed high PTSD rates among adolescents in particularly ranging from 8%-15% due to the civil war and up to 35% due to the 2006 Israeli-Lebanon conflict [12]. More recently, the Beirut Port explosion in 2020, the COVID-19 pandemic, and the country’s economic collapse have further exacerbated Lebanon’s mental health crisis and overwhelmed its healthcare infrastructure [13,14].

Despite the growing burden of mental illness, Lebanon’s mental health system remains under-resourced and fragmented. Mental health accounts for less than 5% of the total health budget, with most resources allocated to inpatient psychiatric care rather than community-based services [15]. Services are concentrated in urban centers, limiting access for rural populations and displaced communities. Moreover, the mental health workforce is insufficient, with only 15.27 providers per 100,000 people [16], and many primary care practitioners lack training in trauma-related disorders [17]. Stigma, limited referral pathways, and cultural barriers further complicate access to appropriate care [18–20].

Displaced families often face additional stressors that contribute to the development or worsening of PTSD, including food insecurity, disrupted social networks, and limited access to stable housing or healthcare. Economic insecurity, marked by unemployment, poverty, and loss of social standing, increases vulnerability to chronic stress and psychiatric disorders [21–23]. At the same time, weakened traditional support systems and persistent social isolation amplify psychological risk [24]. In conflict-affected settings, food scarcity has been linked to elevated symptoms of anxiety, depression, and PTSD [25], while poor housing conditions further contribute to psychological distress [26]. These challenges tend to disproportionately affect vulnerable groups, including women, older adults, and those with limited resources [27–29].

Despite growing awareness of the mental health needs among displaced populations in Lebanon, there is limited research examining how demographic, nutritional, psychosocial, and healthcare access factors interact to predict PTSD risk. The ongoing war in 2024 has further intensified these challenges, underscoring the urgent need for empirical data to inform evidence-based interventions. This study aims to fill this gap by assessing the prevalence and predictors of PTSD symptoms among displaced families in Lebanon amid the current conflict. PTSD symptom severity, measured via the PTSD Checklist (PCL), was analyzed in relation to variables including age, gender, educational attainment, nutritional status (malnutrition and obesity), food security, depression, anxiety, resilience, and access to psychological counselling. The findings intend to inform policies and interventions designed to alleviate PTSD burden and enhance mental health support for displaced communities in Lebanon.

## Methods

### Consent to participate declaration and ethics statement

All participants were informed about the study’s purpose and procedures before their involvement. It was clearly stated that by clicking on the checkbox of the first page of the google form survey, they were providing their written consent to participate. The study was approved by the Ethics Committee of the Modern University for Business and Science under reference number MU-20241106–49. Participation was voluntary, and participants were free to withdraw at any time without consequence.

### Participants

This study is a cross-sectional population survey conducted among 400 participants in various regions of Lebanon, specifically in the Chouf, Aley, Hasbaya, and Rashaya areas, where displaced families from the South and Bekaa areas have relocated. Data were collected from November 10^th,^ 2024, to January 20^th^, 2025, across 20 public schools that provided shelter for displaced families. A total of 20 displaced people in each shelter house or institution were approached by trained researchers and were informed about the purpose of the study. The choice of displaced Lebanese individuals as the subject population is grounded in several reasons, including their high vulnerability and risk of malnutrition and mental health challenges.

The study’s inclusion criteria included adults aged 18 and above residing in Lebanon, with both genders represented. People who had lived abroad or fled the war to other countries were excluded, as were those who did not consent to participate in the study. The participants’ selection did not consider any specific nutritional status or dietary pattern. The sample size was calculated using EpiInfo 7 software for population surveys. Since the approximate frequency of interest (FI) rate was unknown, an expected frequency of 50% was used, along with a 5% margin of error, and a minimum sample size of 384 was calculated (Epiinfo^TM^, version 7.2.5.0).

Every participant was asked to provide their consent, and upon obtaining consent, the trained researchers interviewed participants and completed the online survey to minimize any potential errors in data recording. The questionnaire was offered in English and Arabic and was obtained after the authors’ consent. The participant had the option to complete the anonymous questionnaire in either English or Arabic, according to their preference. The consent was included at the beginning of the survey, and by clicking “I agree to the above”, adult participants were directed to the online survey. The questionnaire clearly stated that participation was voluntary and that declining to participate would have no negative consequences.

### Procedure

The survey consisted of several sections. The first section included sociodemographic variables: parents’ age, gender, education level, family size, current and previous location, duration of displacement, and monthly household income level. The parents’ weight, random plasma glucose, and mid-upper arm circumference (MUAC) were also measured. Subsequent sections of the survey included tools to assess food security, depression, anxiety, PTSD, resilience, as well as access to psychological counseling and food assistance programs.

## Measures

### Anthropometrics

Height was self-reported by participants. Weight was measured using a digital scale by trained researchers after calibration following each data collection. BMI was calculated according to the formula: BMI = weight (kg)/ (height (m)) ^2. BMI categories were defined based on Institute of Medicine (IOM) guidelines: Underweight: BMI < 18.5 kg/m^2^, normal weight: 18.5–24.9 kg/m^2^, overweight: 25.0–29.9 kg/ m^2^, and obese: ≥ 30.0 kg/m^2^ [30].

Mid-Upper Arm Circumference (MUAC) was assessed using a non-stretchable measuring tape on the left arm, at the midpoint between the acromion and olecranon process, with the arm relaxed—a MUAC of **<**23 cm was used as the cut-off for under-nutrition for adults (Van- [31]).

### Biochemical assessment

A finger-prick test was conducted using a portable glucometer to measure capillary blood glucose levels. Standard aseptic techniques were followed for all procedures. Participants were asked to report the fasting hours before testing to differentiate between fasting and random glucose measurements. Blood glucose levels were categorized according to the American Diabetes Association (ADA) criteria. For fasting blood glucose (defined as ≥8 hours of fasting), values <5.6 mmol/L were considered normal, 5.6–6.9 mmol/L were classified as prediabetes, and values ≥7.0 mmol/L were considered indicative of diabetes. For random blood glucose measurements (taken at any time of day, regardless of the last meal), a value ≥11.1 mmol/L accompanied by symptoms of hyperglycemia was considered diagnostic of diabetes [14]. For this study, we classified blood glucose values as either normal (<5.6 mmol/L) or elevated (≥5.6 mmol/L) [32].

#### Food Security:

A food security assessment was conducted using the Arab Family Food Security Scale (AFFSS), which was validated in Lebanon and demonstrated good reliability and psychometric properties [33]. The tool consists of seven questions coded by assigning one point to each positive answer, then summed into a final score that will be categorized into three groups: high food security (score 0–2), low food security (score 3–5), and very low food security (score 5–7). This study grouped the low and very low food security groups into a single category, referred to as “low food security”.

#### Depression:

Depression was screened using the two-item Patient Health Questionnaire (PHQ-2), a validated tool with a sensitivity of 83% and a specificity of 92% at a cut-off value of 3. It consists of two Likert-scale questions with answers ranging from “not at all” to “nearly every day”, coded 0–3 [34]. The Arabic translation of the Patient Health Questionnaire was also previously validated [35].

#### Anxiety:

Anxiety was screened using the Generalized Anxiety Disorder Scale (GAD-2), a validated tool that showed a 76% sensitivity and 81% specificity at a cut-off value of 3. The tool consists of two questions that rate the frequency of feeling nervous, anxious, or on the edge, as well as the inability to stop or control worrying. The answers to each item are coded from 0 to 3, with a maximum total score of 6 per respondent [36]. The Arabic version of the Generalized Anxiety Disorder is validated [37].

#### Post-traumatic stress disorder:

Trauma was screened using the Posttraumatic Stress Disorder Checklist for DSM-5 (PCL-5), a validated tool with good psychometric properties that consists of 20 items with Likert-scale answers coded 0–4 each, and higher scores indicating higher trauma levels [38]. While cut-off values for the PCL-5 vary in the literature, commonly ranging between 31 and 33, a threshold of 31 was adopted in this study to distinguish between probable PTSD and non-PTSD cases [39].The Arabic version of the PCL-5 was validated in the Lebanese population [40].

#### Resilience:

Resilience, defined as the ability to bounce back from stressful situations, was evaluated using the Brief Resilience Scale, a six-item tool with three items worded positively and three worded negatively [41]. Responses are recorded on a Likert scale varying from 1 to note “strongly agree” to 5 corresponding to “strongly disagree,” and the tool is validated in Arabic [42].

The final section consisted of additional questions, including whether any psychological counseling was received during displacement or whether any assistance or support was received regarding food.

## Data analysis

Data was analyzed using statistical software SPSS version 26. Descriptive analysis was employed to represent the variables, utilizing frequencies and percentages to describe the qualitative variables, and means, standard deviations, minimum values, and maximum values to represent the continuous variables. A univariate analysis was performed using an independent samples t-test and ANOVA to assess the differences in PCL-5 scores between the variable groups. Pearson’s correlation coefficient test was used to evaluate the correlation between PCL-5 and other scores used in the study. The variables with a p-value less than 0.2 were entered in the linear regression model (enter method) to assess the factors that predict the PCL-5 score. Another univariate analysis using the chi-square test and Fisher’s exact test was performed to evaluate the differences between the PTSD and no-PTSD groups and was followed by a logistic regression to assess the predictors of the likelihood of PTSD. The significance level of 0.05 was used in this study.

## Results

A total of 400 participants were included in the final analysis (N = 400). The mean age was 42.65 years (SD = 15.39). On average, households consisted of 5.02 members (SD = 2.58), including approximately one child under the age of 18 (mean = 0.99, SD = 1.61). The average duration of displacement was 55.71 days (SD = 33.82). Approximately three-quarters of the participants were females (n = 297, 74.3%). More than half had either primary education (n = 145, 36.3%) or no formal education (n = 109, 27.3%). The majority (n = 333, 83.3%) reported a monthly household income below 700 USD.

While 168 participants (42.0%) reported receiving food-related assistance or support, more than half (n = 231, 57.8%) had a high food security score. Most participants had normal blood glucose levels (n = 365, 91.3%).

The mean mid-upper arm circumference (MUAC) was 30.19 cm (SD = 5.85), with the majority (n = 371, 92.8%) falling within the normal range. Additionally, nearly one-fifth of participants (n = 84, 21.0%) were classified as obese (Table 1).

**Table 1:** Baseline characteristics of the study population (N = 400).

		Frequency	Percent
**Gender**	Male	103	25.8
	Female	297	74.3
**Household members number**	Mean (SD)	5.02 (2.58)	
**Household children under 18**	Mean (SD)	0.99 (1.61)	
**Displacement duration (days)**	Mean (SD)	55.71 (33.82)	
**Education level**	No formal education	109	27.3
	Primary education	145	36.3
	Secondary education	58	14.5
	Tertiary education	88	22.0
**Monthly household income level**	<700$	333	83.3
	700-1500$	57	14.3
	1500-3000$	7	1.8
	≥3000$	3	0.8
**Assistance or support regarding food**	No	232	58.0
	Yes	168	42.0
**MUAC, cm**	Mean (SD)	30.19 (5.85)	
	Normal (≥23 cm)	371	92.8
	Malnutrition (<23 cm)	29	7.3
**Arab Food Security Score**	Mean (SD)	400 (2.41)	
	High food security (Score 0–2)	231	57.8
	Low food security (Score 3–5)	105	26.3
	Very low food security (Score 5–7)	64	16.0
**Blood glucose value**	Mean (SD)	110.09 (33.89)	
	Normal (Fasting<5.6 mmol/L; Random<11.1 mmol/L)	365	91.3
	High	35	8.8
**BMI, kg/m** ^**2**^	Mean (SD)	26.41 (5.44)	
**Obesity, BMI ≥ 25 kg/m** ^**2**^	No	316	79.0
	Yes	84	21.0

**Fig 1:**
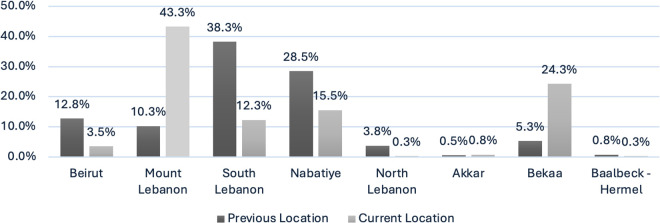
Displacement patterns. Distribution of previous and current locations among displaced families in Lebanon. Dark gray bars represent previous location; light gray bars represent current location.

The displacement pattern revealed a marked decrease in the proportion of residents living in Beirut (from 12.8% before the war to 3.5% during the war), South Lebanon (from 38.3% to 12.3%), and Nabatiye (from 28.5% to 15.5%). On the contrary, the % of residents increased in Mount Lebanon and Bekaa to 43.3% and 24.3% respectively (Fig 1).

The mental health status of displaced individuals is summarized in Table 2. A relatively high mean PCL-5 score of 40.53 (SD = 20.75) was observed, with 259 participants (64.8%) screening positive for PTSD.As for resilience, a mean score of 3.08 (SD = 0.42) was noted among the displaced, the majority (240, 67.5%) having a normal resilience, and almost one third showing low resilience (124, 31%). Regarding depression, the mean PHQ-2 score was 3.13 (SD = 1.82), with 57.5% of participants meeting the criteria for major depressive disorder. Similarly, 57.0% screened positive for GAD, with a mean GAD-2 score of 3.15 (SD = 1.91). Despite these high rates of psychological distress, only 113 participants (28.3%) reported receiving psychological counseling during displacement (Table 2).

**Table 2:** Mental health characteristics of the displaced families.

Mental health variables		Frequency	Percent
**PTSD**	PCL5 score Mean (SD)	40.53 (20.75)	
	No PTSD (PCL5 < 31)	141	35.3
	PTSD (PCL5 Score ≥ 31)	259	64.8
**PHQ2**	PHQ2 score Mean (SD)	3.13 (1.82)	
	Non-depressed (< 3)	170	42.5
	Depressed (≥ 3)	230	57.5
**GAD2**	GAD2 score Mean (SD)	3.15 (1.91)	
	No anxiety (< 3)	172	43.0
	Anxiety (≥ 3)	228	57.0
**BRS**	BRS Score Mean (SD)	3.08 (0.42)	
	Low (BRS < 3.00)	124	31.0
	Normal (3.00 ≤ BRS ≤ 4.30)	270	67.5
	High (BRS > 4.30)	6	1.5
**Have you received any psychological counseling during your displacement**	No	287	71.8
	Yes	113	28.3

### PCL-5 score predictors

A linear regression model was performed and presented in Table 3 with F (14, 395) = 26.81, p < 0.001, with an adjusted R^2^ of 47.6%. Several factors were associated with PTSD. Higher levels of depression (B = 2.853, p < 0.001), anxiety (B = 2.997, p < 0.001), and food insecurity (B = 0.803, p = 0.042) were associated with increased PCL-5 scores, indicating greater PTSD symptom severity.

**Table 3:** Linear regression model of factors predicting PTSD using PCL5 Score.

	Unstandardized Coefficients		Standardized Coefficients	t	Sig.
	B	Std. Error	Beta		
**(Constant)**	50.596	8.441		5.994	<0.001***
**Age, years**	0.051	0.053	0.038	0.959	0.338
**Gender**	2.665	1.823	0.056	1.462	0.144
**Family Size, number of people in family**	-0.261	0.301	-0.032	-0.866	0.387
**Number of children under 18**	0.911	0.493	0.070	1.848	0.065
**Education level**	-2.054	0.744	-0.109	-2.760	0.006**
**Length of displacement, days**	-0.115	0.023	-0.187	-5.007	<0.001***
**Monthly household income level**	-0.718	1.683	-0.017	-0.427	0.670
**Obesity (BMI ≥ 30 kg/m**^**2**^)	0.998	1.914	0.020	0.521	0.602
**Malnutrition (MUAC < .23 cm)**	-5.599	2.975	-0.070	-1.882	0.061
**AFFS**	0.803	0.394	0.088	2.038	0.042*
**PHQ-2**	2.853	0.598	0.250	4.769	<0.001***
**GAD-2**	2.997	0.570	0.276	5.253	<0.001***
**BRS**	-8.210	1.922	-0.165	-4.272	<0.001***
**Receiving any psychological counseling during displacement**	2.394	1.712	0.052	1.398	0.163

*p <.05. **p <.01. ***p <.001

Conversely, higher levels of resilience (B = -8.21, p < 0.001), higher educational attainment (B = -2.05, p = 0.006), and longer duration of displacement (B = -0.11, p < 0.001) were significantly associated with lower PCL-5 scores, suggesting a protective effect against PTSD symptoms.

### PTSD Predictors: Clinical perspective

Several factors were compared between participants with and without PTSD (based on a PCL-5 cutoff score of 31) and are presented in Table 4, using Chi-square or Fisher’s Exact tests as appropriate. The PTSD group included a significantly higher proportion of females (68.4%, *p* = 0.01), individuals with no formal education (80.7%, *p* < 0.001), and households with a monthly income below 700 USD (68.8%, *p* = 0.002), compared to the non-PTSD group.

**Table 4:** Comparison of participants’ characteristics across the PTSD groups*.

Variable	Groups	PTSD groups				p-value
		No PTSD		PTSD (PCL5 > 31)		
		N/ Mean	%/SD	N/ Mean	%/SD	
**Gender**	Male	47	45.6%	56	54.4%	0.01*
	Female	94	31.6%	203	68.4%	
**Education Level**	No formal education	21	19.3%	88	80.7%	< 0.001***
	Primary education	53	36.6%	92	63.4%	
	Secondary education	26	44.8%	32	55.2%	
	Tertiary education	41	46.6%	47	53.4%	
**Monthly Household Income Level**	<700$	104	31.2%	229	68.8%	0.002**
	700-1500$	33	57.9%	24	42.1%	
	1500-3000$	3	42.9%	4	57.1%	
	≥3000$	1	33.3%	2	66.7%	
**AFFS**		1.70	2.13	2.80	2.28	< 0.001***
**Food Security**	High food security (Score 0–2)	102	44.2%	129	55.8%	< 0.001***
	Low food security (Score 3–5)	23	21.9%	82	78.1%	
	Very low food security (score???)	16	25.0%	48	75.0%	
**PHQ-2**		2.12	1.49	3.68	1.74	< 0.001***
**Depression**	Normal (<3)	86	50.6%	84	49.4%	< 0.001***
	Major depressive disorder (≥3)	55	23.9%	175	76.1%	
**GAD-2**		1.95	1.54	3.81	1.77	< 0.001***
**Anxiety**	Normal (<3)	98	57.0%	74	43.0%	< 0.001***
	Generalized Anxiety Disorder (≥3)	43	18.9%	185	81.1%	
**BRS**		3.26	0.49	2.98	0.33	< 0.001***
**Resilience**	Low (BRS < 3.00)	31	25.0%	93	75.0%	< 0.001***
	Normal (3.00 ≤ BRS ≤ 4.30)	104	38.5%	166	61.5%	
	High (BRS > 4.30)	6	100.0%	0	0.0%	
**Psychological counseling during displacement**	No	113	39.4%	174	60.6%	0.006**
	Yes	28	24.8%	85	75.2%	

p-values for continuous variables were calculated using Independent Samples t-tests; categorical variables were compared using Chi-Square Test or Fisher’s Exact Test. *p <.05. **p <.01. ***p <.001

No significant differences were observed between the groups in terms of age, family size, number of children under 18 years old, duration of displacement, obesity, malnutrition, blood glucose levels, or receipt of food assistance.

However, food security status was significantly lower among those with PTSD, with 78.1% of participants in the PTSD group classified as having low food security and 75.0% with very low food security (*p* < 0.001). In addition, the PTSD group had significantly higher rates of major depressive disorder (76.1%, *p* < 0.001), generalized anxiety disorder (81.1%, *p* < 0.001), and reported receiving psychological counseling (75.2%, *p* = 0.006).

The mean depression and anxiety scores were also significantly elevated in the PTSD group, at 3.68 and 3.81, respectively (*p* < 0.001 for both). Moreover, three-quarters (75.0%) of participants with low resilience were identified in the PTSD group, and none of those with high resilience screened positive for PTSD (*p* < 0.001). The overall mean resilience score was significantly lower among participants with PTSD (2.98) compared to those without PTSD (3.26; *p* < 0.001), as shown in Table 4.

### Binary logistic regression analysis of PTSD predictors

A binary logistic regression was conducted to identify factors associated with the likelihood of screening positive for PTSD among displaced individuals (Table 5). The overall model was statistically significant, as indicated by the Omnibus Test of Model Coefficients (*p* < 0.001), and showed good fit (Hosmer and Lemeshow Test, *p* = 0.324). The model explained 41.9% of the variance in PTSD status (Nagelkerke R^2^ = 0.419) and correctly classified 76.5% of cases. Using Enter method, a total of 11 predictors were included in the model, of which five were statistically significant: education level, psychological counseling during displacement, anxiety, depression, and resilience as shown in the table below.

**Table 5:** Logistic regression model to assess predictors of PTSD.

	B	S.E.	p-value	Odds Ratio (OR)	95% Confidence Interval for OR	
					Lower	Upper
**Age, years**	0.009	0.009	0.312	1.009	0.992	1.026
**Gender, female**	-0.066	0.296	0.825	0.937	0.524	1.673
**Education level**			0.014*			
**No formal education**	1.010	0.406	0.013*	2.744	1.239	6.077
**Primary education**	-0.126	0.352	0.721	0.882	0.442	1.757
**Secondary education**	0.388	0.430	0.367	1.474	0.635	3.423
**Monthly household income level**	-0.164	0.256	0.522	0.849	0.514	1.402
**Length of displacement, days**	-0.007	0.004	0.067	0.993	0.985	1.001
**Receiving any psychological counseling during displacement, yes**	-0.643	0.303	0.034***	0.526	0.291	0.951
**AFFS**	-0.074	0.194	0.704	0.929	0.636	1.358
**Obesity (BMI ≥ 30 kg/m** ^**2**^ **), yes**	-0.084	0.326	0.796	0.919	0.485	1.742
**GAD2**	0.449	0.099	<0.001***	1.567	1.290	1.904
**PHQ2**	0.265	0.101	0.009**	1.303	1.069	1.590
**BRS**	-1.248	0.345	<0.001***	0.287	0.146	0.565
**Constant**	3.108	1.313	0.018*	22.374		

*p <.05. **p <.01. ***p <.001

Participants with no formal education had significantly higher odds of PTSD compared to those with some education (OR = 2.77; *p* = 0.013). Higher anxiety (GAD-2 score) and depression (PHQ-2 score) were also associated with an increased likelihood of PTSD, with odds ratios of 1.576 (*p* < 0.001) and 1.303 (*p* = 0.009), respectively.

In contrast, greater resilience was strongly protective, with a significantly reduced likelihood of PTSD (OR = 0.287, *p* < 0.001). Additionally, receiving psychological counseling during displacement was associated with a reduced risk of PTSD, cutting the odds nearly in half (OR = 0.526, *p* = 0.034), as shown in Table 5.

## Discussion

This is the first study conducted in Lebanon assessing PTSD and its associated factors in internally displaced individuals following the 2024 war. The pervasive impact of PTSD remains a significant public health concern in low- to middle-income countries, particularly those grappling with ongoing political and economic instability, such as Lebanon [43]. Our findings revealed that mental health issues were highly prevalent, with 64.8% of participants screening positive for PTSD. This significantly surpasses the 29.3% prevalence during the 2006 war, highlighting a concerning exacerbation of mental distress [44]. This increased burden might be strongly associated with the high comorbidity of major depressive disorder (57.5% meeting criteria) and generalized anxiety disorder (57.0% screening positive) observed in this population.

Both linear and binary logistic regression analyses established depression (B = 2.853, OR = 1.303) and anxiety (B = 2.997, OR = 1.576) as strong predictors of PTSD agreeing with previous literature that these conditions often coexist with PTSD and reinforce each other [45, 46]. Moreover, studies from Lebanon and the MENA region have similarly shown the close relation between PTSD, depression, and anxiety, particularly in populations exposed to traumatic events such as conflict and displacement. For example, Farhood et al. [47] reported a 23.4% prevalence of PTSD and major depressive disorder co-occurrence among South Lebanese civilians, with depression being strongly linked to PTSD symptoms [47]. Similarly, another study that looked at 5,140 traumatized Arabic-speaking individuals from the MENA region concluded that 43.8% of participants exhibited high general PTSD symptoms that were accompanied by elevated levels of depressive and anxiety symptoms [48]. This highlights the importance of addressing co-occurring mental health conditions when screening and applying interventions for PTSD.

The demographic profile of participants—predominantly female (74.3%), with low educational attainment (63.6% having only primary or no formal education), and limited household income (83.3% earning below $700/month)—reflects the typical characteristics of displaced populations, where these factors amplify the psychological burden of displacement [49]. Both linear and logistic regression analyses revealed that higher educational attainment was significantly associated with lower PTSD scores (B = -2.054), while having no formal education was linked to significantly higher odds of PTSD (OR = 2.77), highlighting the protective role of education as a resilience factor. Additionally, females represented a considerably larger portion of the PTSD group (68.4%, p = 0.01), emphasizing the need for gender-sensitive mental health interventions. The heightened vulnerability of females to PTSD is well-documented, and some studies suggest that females are twice as likely to develop PTSD as males [50]. This may be attributed to a combination of biological, psychological, and sociocultural factors that makes them more vulnerable to trauma-related disorders [51–53]

Conversely, individuals with high resilience scores were less likely to exhibit PTSD symptoms, aligning with previous studies suggesting that having resilient attributes enables individuals to cope better and mitigate the impact of traumatic events [54,55]. The mean resilience score was significantly lower among those with PTSD (2.98 vs. 3.26 without PTSD, p < 0.001), further emphasizing its protective role. Furthermore, a linear regression analysis indicated that a longer duration of displacement (mean 55.71 days) was associated with lower PTSD scores (B = -0.115). While chi-square analysis did not reveal a significant difference in displacement duration between the PTSD groups, this subtle association in the linear model may reflect a gradual adaptation or the development of coping strategies over time, a phenomenon observed in other long-term displacement contexts [56]. This insight requires further longitudinal investigation to better understand the psychological adjustments in such events.

Food insecurity emerged as a significant potential predictor for PTSD, which is usually overlooked. Despite 42.0% of participants reporting some form of food assistance, a substantial 42.3% (26.3% low, 16.0% very low) reported food insecurity, and this was significantly associated with increased PTSD symptom severity (B = 0.803). This is expected, since uncertainty around access to food has been shown to amplify stress and contribute to emotional trouble during traumatic events [57]. This underscores the critical, often overlooked, mental health dimension of food security. Interestingly, almost half of the participants experienced some degree of food insecurity, with the mean BMI being overweight (26.41 kg/m^2^), while 21.0% were classified as obese. Moreover, a normal MUAC measurement (mean 30.19 cm, 92.8% within the normal range) was identified for this population, with only 7% being classified as malnourished. This paradox may be due to the relatively short duration of displacement experienced by this group approximately 56 days) which may not have been sufficient to promote malnutrition. For example, a study of Syrian refugees in Lebanon who had been displaced for over 5 years found a malnutrition prevalence of 22.1**%** of children under five [58]. Similarly, a study done on individuals in Ethiopia who have been internally displaced for over two years has shown a 30% prevalence of moderate to severe malnutrition [59]. This suggests that malnutrition may become more apparent in longer-term displacement settings. Another explanation for this paradox is the concept of the “double burden” of malnutrition, a common phenomenon in humanitarian settings where individuals tend to rely on high-calorie, dense foods because they are less expensive and more readily available [60,61]. This could also lead to long-term nutritional deficiencies alongside mental health challenges.

Perhaps one of the most compelling insights from this study is the limited access these individuals had to mental health services. Our study found that fewer than 30% of participants reported receiving any psychological support during displacement. Among those who did receive counseling, there was a significantly reduced likelihood of PTSD (OR = 0.526), decreasing the odds of PTSD by nearly half. Conversely, individuals with PTSD were significantly more likely to have not received counseling (60.6% vs. 39.4% in the non-PTSD group, p = 0.006), and those who did were substantially less likely to meet PTSD criteria. Our findings agreed with other studies that showed the heavy burden of mental health among internally displaced individuals [62,63]. For example, A study in Beirut’s Shatila camp revealed that individuals who did not receive any mental health care were 58% more likely to develop mental illness [64]. This is critical not because of the immediate effect of the absence of mental health aid during displacement, but because of the detrimental long-term impacts this exerts. A study examining survivors of WWII in Germany found that forced displacement had long-lasting psychological consequences, including persistent PTSD and poorer quality of life even decades later [65]. This finding powerfully reinforces the critical importance of integrating mental health care into humanitarian aid efforts during traumatic events, as it can substantially aid recovery among those affected and avoid long-term effects of PTSD [66].

While this study provides crucial insights, it has several limitations, including its reliance on self-reported screening tools for mental health variables, which do not replace clinical diagnosis and may be subject to bias. Additionally, the cross-sectional design prevents us from establishing causality. Finally, the sample may not fully represent the broader displaced population, particularly those who fled the country or are residing in areas inaccessible to aid.

Despite its limitations, this study is the first to investigate predictors of PTSD in Lebanon during the 2024 conflict, offering novel insights into the mental health challenges faced by displaced populations. Our findings revealed a high prevalence of psychological distress, with PTSD significantly associated with factors such as food insecurity, anxiety, depression, low educational attainment, and limited access to mental health services. In contrast, resilience and longer duration since displacement were found to be protective factors.

These results highlight the urgent need for integrated humanitarian responses that go beyond meeting basic needs to include mental health support and address the underlying drivers of psychological distress. Programs should prioritize both immediate relief and long-term psychological resilience, particularly by strengthening access to mental health care and improving food security. Given Lebanon’s ongoing political and social instability, early interventions are critical.
