# Correction: Predictors of posttraumatic stress disorder among displaced families in Lebanon: A cross-sectional study

**DOI:** 10.1371/journal.pgph.0005905

**Published:** 2026-01-28

**Authors:** Paula Hage Boutros, Samah Hachem, Bachir Atallah, Malak Awada, Rola Bou Serhal

The fifth author’s initials appear incorrectly in the citation. The correct citation is: Boutros PH, Hachem S, Atallah B, Awada M, Bou Serhal R. (2025) Predictors of posttraumatic stress disorder among displaced families in Lebanon: A cross-sectional study. PLOS Glob Public Health 5(10): e0005280. https://doi.org/10.1371/journal.pgph.0005280.

The ORCID iDs are missing for the fifth author. Please see the authors’ respective ORCID iD here:

- Author Rola Bou Serhal’s ORCID iD is: 0000-0002-1132-5834

(https://orcid.org/0000-0002-1132-5834)
